# Screening and Molecular Docking of Novel Benzothiazole Derivatives as Potential Antimicrobial Agents

**DOI:** 10.3390/antibiotics9050221

**Published:** 2020-04-29

**Authors:** Mohamed A. Morsy, Enas M. Ali, Mahmoud Kandeel, Katharigatta N. Venugopala, Anroop B. Nair, Khaled Greish, Mahmoud El-Daly

**Affiliations:** 1 Department of Pharmaceutical Sciences, College of Clinical Pharmacy, King Faisal University, Al-Ahsa 31982, Saudi Arabia; kvenugopala@kfu.edu.sa (K.N.V.); anair@kfu.edu.sa (A.B.N.); 2 Department of Pharmacology, Faculty of Medicine, Minia University, El-Minia 61511, Egypt; 3 Department of Biological Sciences, College of Science, King Faisal University, Al-Ahsa 31982, Saudi Arabia; eabdelkader@kfu.edu.sa; 4 Department of Botany and Microbiology, Faculty of Science, Cairo University, Cairo 12613, Egypt; 5 Department of Biomedical Sciences, College of Veterinary Medicine, King Faisal University, Al-Ahsa 31982, Saudi Arabia; mkandeel@kfu.edu.sa; 6 Department of Pharmacology, Faculty of Veterinary Medicine, Kafrelsheikh University, Kafr El-Sheikh 33516, Egypt; 7 Department of Biotechnology and Food Technology, Durban University of Technology, Durban 4000, South Africa; 8 Department of Molecular Medicine, Princess Al-Jawhara Centre for Molecular Medicine, School of Medicine and Medical Sciences, Arabian Gulf University, Manama 329, Bahrain; khaledfg@agu.edu.bh; 9 Department of Pharmacology & Toxicology, Faculty of Pharmacy, Minia University, El-Minia 61511, Egypt; eldaly_m@mu.edu.eg

**Keywords:** antimicrobial, benzothiazole derivatives, molecular docking, dihydroorotase, dimorphic transition

## Abstract

The burden of antibiotic resistance necessitates a continued search for new antimicrobials. We evaluated the antimicrobial activities of novel benzothiazoles synthesized by our group. Antibacterial activity was evaluated in vitro in *Staphylococcus aureus*, *Bacillus subtilis*, and *Escherichia coli*, while the antifungal activity was tested in *Candida albicans* and *Aspergillus niger*, and expressed as the minimum inhibitory concentration (MIC; µg/mL). MIC values of benzothiazole compounds ranged from 25 to 200 µg/mL. Compounds 3 and 4 gave high antibacterial and moderate antifungal activities, while 10 and 12 showed moderate activity against all tested organisms. In addition, some benzothiazole compounds significantly suppressed the activity of *Escherichia coli* dihydroorotase and inhibited the dimorphic transition of *Candida albicans*. Moreover, the active benzothiazole compounds induced DNA and protein leakage in *Aspergillus niger* spores. Molecular interactions of benzothiazole derivatives with dihydroorotase revealed the formation of hydrogen bonds with the active site residues LEU222 or ASN44. Strong hydrophobic interactions of the bulky thiazole and naphthalene rings at the entrance to the active site might interfere with the access of substrates to their binding sites, which results in dihydroorotase inhibition. Thus, inhibition of dihydroorotase might contribute to the observed antimicrobial actions of these compounds.

## 1. Introduction

Microbial resistance is a problem that arises due to the widespread use of antimicrobial drugs in human, veterinary, and agricultural applications [1]. Microbes gain resistance through many mechanisms such as prevention of access to the target and changes in antibacterial targets by mutation as well as direct modification of drugs [2]. The increased incidence of microbial resistance to currently available antimicrobial agents and its burden on global healthcare necessitate the continued research and development in the field of anti-infective drugs [3,4]. Thus, the continued search for new antibacterial and antifungal drugs is highly encouraged.

The benzothiazole nucleus, with a variety of modifications, has demonstrated a wide range of pharmacological properties including antimicrobial [5], anti-HIV [6], anthelmintic [7], antidiabetic [8], anticonvulsant [9], larvicidal [10], antimalarial [11], and anticancer activities [12]. In addition, the potential antimicrobial activity of benzothiazole derivatives was previously illustrated [13,14,15,16,17]. The results of these studies concluded that the antimicrobial activity of benzothiazole derivatives can be attributed to its ability to interact with several cellular targets including *Staphylococcus* (*S.*) *aureus* DNA gyrase [18,19,20], *Escherichia* (*E.*) *coli* dihydropteroate synthase [21], *Plasmodium* dihydrofolate reductase [22], and, recently, *E. coli* dihydroorotase [23]. Thus, the aim of the current study was to test the antimicrobial potential of benzothiazole derivatives newly designed and synthesized by our research group [24] and to investigate their possible mechanism of action. To achieve this aim, we followed two approaches: an experimental in vitro approach by testing the inhibitory effect of such compounds on microbial growth and an *in silico* approach to determine the ability of the tested compounds to bind the specified targets using computer-assisted docking studies.

## 2. Results

### 2.1. Antimicrobial Activity

**Figure 1:**
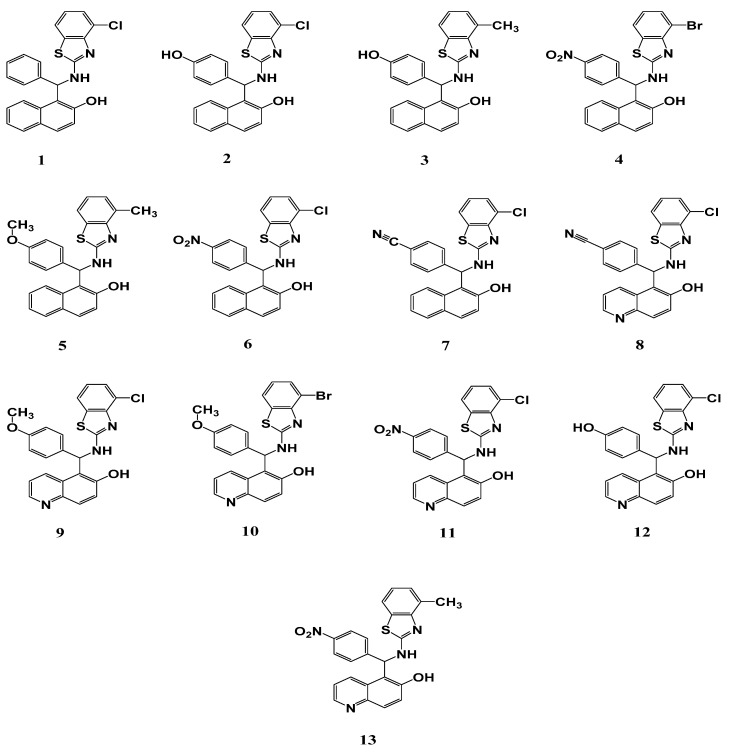
Chemical structure of benzothiazole derivatives screened for antimicrobial properties.

The chemical structures of the studied benzothiazole derivatives are shown in Figure 1. The results of the antimicrobial activity study, expressed as the diameter (mm) of the inhibition zone (IZD), are shown in Table 1. Some of the tested compounds exerted moderate to good in vitro antibacterial activity against the tested organisms, as indicated by IZD of 6–27 mm (Table 1). The standard antibiotic kanamycin showed the highest inhibitory activity against the three tested bacterial strains as well as the highest potency (Table 1). Among the tested benzothiazole derivatives, compounds 3 and 4 showed the most significant inhibitory activity, especially against *E. coli*. The minimum inhibitory concentration (MIC) values for the different compounds ranged from 50 to 200, 25 to 200, and 25 to 100 µg/mL for *S. aureus*, *Bacillus* (*B.*) *subtilis*, and *E. coli*, respectively. On the other hand, the minimum bactericidal concentration (MBC) values ranged from 100 to 400, 50 to 400, and 50 to 200 µg/mL, respectively, for these bacteria (Table 1). The antibacterial potential of the different benzothiazole compounds against *E. coli* varied significantly, as illustrated by the higher activity of compounds 3 and 4 (IZD: 27 and 25, respectively), while compounds 5, 10, and 12 showed moderate effects (IZD: 18, 12, and 19 mm, respectively). However, compound 2 displayed only weak antibacterial activity against *E. coli*. On the contrary, compounds 1, 6, 7, 8, 9, 11, and 13 like dimethyl sulfoxide (DMSO) failed to display any antibacterial activity against *E. coli*. Nonetheless, compounds 1, 2, and 5 showed moderate antibacterial activity against the used Gram-positive organisms.

The antifungal activity of different benzothiazole compounds was evaluated against *Candida* (*C.*) *albicans* and *Aspergillus* (*A.*) *niger*. The standard antifungal agent fluconazole showed the highest inhibitory activity against the two fungal strains used in the current experiment. The results illustrated in Table 1 summarize the antifungal activity of the 13 benzothiazole compounds. These results indicated that compounds 3, 4, 10, and 12 exhibited moderate inhibitory effects on fungal growth, while compound 2 displayed weak antifungal activity against both tested fungi. Moreover, compound 1 was weakly active against *A. niger* only. On the other hand, compounds 5, 6, 7, 8, 9, 11, and 13, like DMSO, lacked any antifungal activity.

### 2.2. Effect of Benzothiazole Compounds on the Activity of E. coli Dihydroorotase

Dihydroorotase is an enzyme essential for cellular pyrimidine synthesis. The effectiveness of the tested compounds as inhibitors of *E. coli* dihydroorotase was evaluated and the results are shown in Table 2. The activity of the enzyme was suppressed variably as a result of its treatment with the 13 benzothiazole compounds. Of the tested compounds, compound 3 was the most effective since it reduced the specific activity of dihydroorotase to 45 nmol/min/mg protein, which was followed by compound 4 (60 nmol/min/mg protein). On the other hand, compounds 10, 11, and 12 were moderately active, while compounds 1, 2, 5, 6, 7, 8, 9, and 13 showed only a little or no activity at all. 

### 2.3. Effect of Benzothiazole Compounds on Dimorphic Transition of C. Albicans

**Figure 2:**
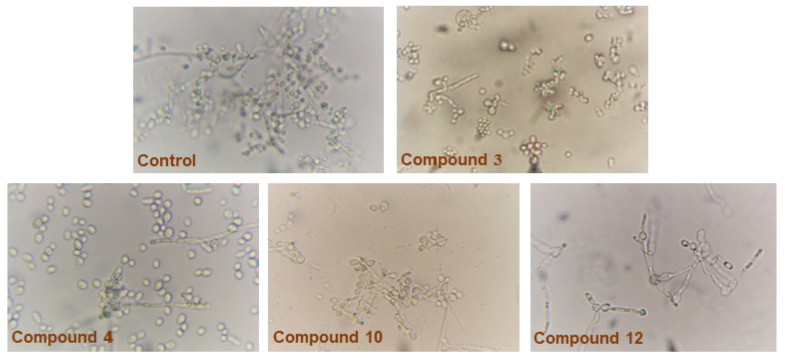
Effect of benzothiazole compounds on dimorphic transition of *Candida albicans*. Strong inhibition of dimorphic transition was observed for compounds 3 and 4 (most cells are in yeast form).

Data in Table 3 and Figure 2 show the effect of the 13 benzothiazoles on the morphogenic transition of *C. albicans*. Untreated controls displayed significant hyphal growth after 6 h, while this ability of *C*. *albicans* to transform morphologically was repressed by some of the tested benzothiazoles to variable extents. In addition, inhibition of dimorphism was established at concentrations significantly lower than the respective MIC values. For example, compound 3, which displayed the highest potency against *C. albicans* (MIC: 25 µg/mL), inhibited dimorphism at 12.5 µg/mL. On the other hand, compound 10, which displayed MIC at 100 µg/mL, inhibited the dimorphism at 50 µg/mL. Similarly, compounds 4 and 12 inhibited dimorphism at 25 µg/mL. On the other hand, compounds 6, 7, 8, 9, 11, and 13, like DMSO, failed to appreciably inhibit dimorphism, where most of the *C. albicans* cells were in filamentous form.

### 2.4. Determination of DNA and Protein Leakage

**Figure 3:**
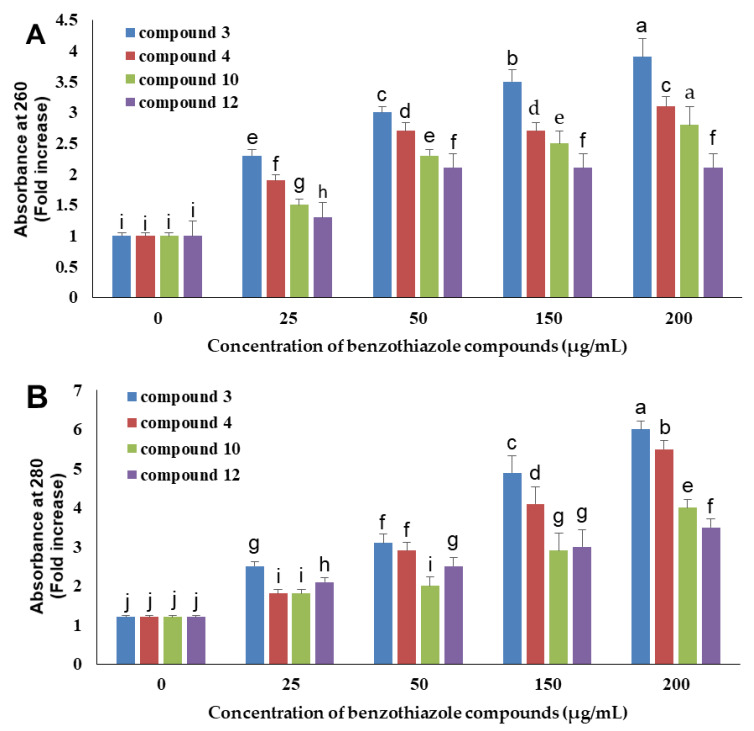
Different benzothiazole compounds at different concentrations caused the leakage of DNA (**A**) and protein (**B**) from *Aspergillus niger* spores. Data represent the means ± standard deviation of three independent replicates, and bars with the same letters are not significantly different from each other, according to ANOVA followed by Duncan’s multiple range tests at *p* < 0.05.

As illustrated in Figure 3, the results of the current study showed that benzothiazole compounds having antifungal activity were able to induce DNA and protein leakage from *A. niger* spores. Compound 3 demonstrated the best activity in this experiment, which was followed by 4, 10, and 12.

Compound 3 displayed the best activity in this experiment, which was followed by compounds 4, 10, and 12. Furthermore, the leakage of DNA and proteins was concentration-dependent (Figure 3). In the presence of compound 3 at 25, 50, 150, and 200 µg/mL, the optical density at 260 nm (OD_260_) values increased by 2.3, 3.0, 3.5, and 3.9 folds, respectively (Figure 3A), while the OD_280_ values increased by 2.5, 3.1, 4.9, and 6.0 folds, respectively (Figure 3B).

### 2.5. Interactions of Benzothiazole Compounds with E. coli Dihydroorotase

**Figure 4:**
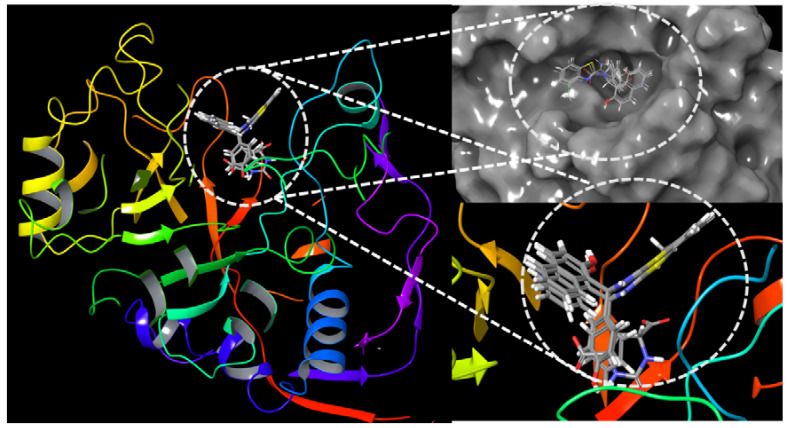
The docking site of benzothiazole compounds with *Escherichia coli* dihydroorotase.

**Figure 5:**
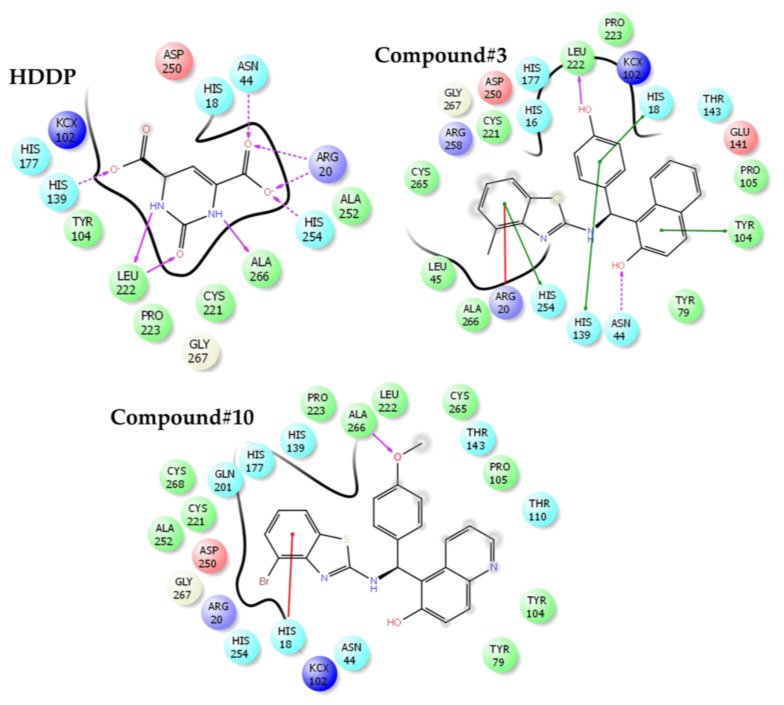
The binding mode and ligand interactions of 2-oxo-1,2,3,6-tetrahydropyrimidine-4,6-dicarboxylic acid (HDDP) and benzothiazole compounds 3 and 10. The stacking interactions are shown in green or red sticks. Hydrogen bonds are in purple arrows. The direction of the arrow is toward the hydrogen receptor.

The results of the docking run of biologically active benzothiazole compounds with *E. coli* dihydroorotase are summarized in Table 4. The benzothiazole compounds show a Y-shaped conformation where the benzene ring derivatives of the benzothiazole compounds lodges into the active site cavity hosting 2-oxo-1,2,3,6-tetrahydropyrimidine-4,6-dicarboxylic acid (HDDP) (Figure 4). The co-crystalized ligand, HDDP, showed the highest docking score of −7.37. On the other hand, the benzothiazole compounds showed docking scores ranging between −2.54 and −5.02, where compounds 3 and 10 showed the highest docking scores. In terms of ligand efficiency parameters, compounds 3, 4, and 10 showed approximately 29% of HDDP ligand efficiency. The obtained lower ligand efficiency and docking scores might be attributed to the loss of essential hydrogen bonds and the increased hydrophobic interactions of the benzothiazole compounds. The hydrophobic interactions displayed by the benzothiazoles were 6.5–11.4 times higher than HDDP. The hydrogen bonding was reduced by a value of 35–80% (Figure 5).

## 3. Discussion

The aim of this study was to test the antimicrobial activity of some newly introduced benzothiazole derivatives and to identify the possible mechanism(s) underlying their activities. The synthesis and characterization of the benzothiazoles used in the current study were recently reported by our research group [24], which showed the promising value of these compounds as antitubercular drugs. In the current study, four of the tested compounds (3, 4, 10, and 12) showed promising activity against the studied pathogenic bacterial models: *S. aureus, B. subtilis*, and *E. coli* as well as moderate antifungal effects against two fungi: *C. albicans* and *A. niger*. The best antibacterial activity results were achieved with compound 3, which was highly active against all tested bacterial species followed by compound 4. On the other hand, compounds 10 and 12 were moderately active against the tested bacterial strains. Since none of the studied compounds showed high antifungal activity in our results, no further docking studies for these compounds with fungal targets were carried out.

Benzothiazole compounds display a broad-spectrum biological activity [5,6,8,11,14,17,20,21,22,23,24]. Thus, they usually act as an important guide frame and parent skeleton, which plays very important roles in medicinal chemistry and agrochemicals. However, to the best of our knowledge, few reports on antimicrobial activity of benzothiazole derivatives against pathogenic fungi and bacteria have been published [14,18,23]. Benzothiazole-based antibacterial compounds bind to different biological targets in bacterial cells and have been shown to be inhibitors of enzymes that are important for essential processes in the bacterial cells such as cell wall synthesis, cell division, and DNA replication, or biosynthesis of essential compounds [22,25,26]. Several benzothiazole-based compounds have been shown to interact with molecular targets in *Mycobacterium tuberculosis* [6,18,24,25]. Some benzothiazole compounds act as inhibitors of type II topoisomerases, which are enzymes that catalyze changes in DNA topology by breaking and rejoining double-stranded DNA [27,28].

Our results were parallel with those of Padalkar and his colleagues [14] who reported variable inhibitory effects on the growth of *E. coli*, *S. aureus*, *C. albicans*, and *A. niger* by some synthesized benzothiazole derivatives. Similarly, Luo et al. [29] found that some benzothiazole compounds exhibited significant antifungal activities against some plant pathogens where some compounds inhibited the growth of *Fusarium solani* with IC_50_ of 4.34–17.61 µg/mL, which were more potent than that of the positive control; hymexazol (IC_50_ of 38.92 µg/mL). Moreover, a series of novel 4-substituted and 5-substituted methyl sulfonyl benzothiazole derivatives showed promising antimicrobial activity at an MIC range of 4–50 µg/mL against selected bacterial as well as fungal species [13]. Similar results were obtained by others [30,31] that support our findings in the current study showing that benzothiazole derivatives are active against both Gram-positive and Gram-negative bacteria. However, the results obtained by Saeed et al. [32] indicated that some benzothiazole compounds possessed a broad spectrum of antimicrobial activity, but, unlike our results in the current study, they showed higher activity against fungi than bacteria. The structure–activity relationship in that study proposed that electronic factors in benzothiazole rings had a great effect on the antimicrobial activity of such compounds [32].

In order to study the possible mechanism(s) of antimicrobial activity of benzothiazole compounds in the current study, we tested the effect of the active antifungal candidates on cellular permeability of *A. niger* spores by monitoring DNA and protein leakage. The results showed that these compounds increased leakage of both DNA and proteins from *A. niger* spores in a concentration-dependent manner. Our results went parallel with those of Singh et al. [33] who conclude that a series of benzothiazoles could disrupt the integrity of the plasma membrane, which leads to cell content leakage. Moreover, the same group [34] illustrated the ability of some benzothiazole compounds to alter cellular membrane potential via pore formation and/or destabilization, which ultimately altered membrane integrity. Therefore, in the current study, the results highlight the possibility that the antimicrobial activity of compounds 3, 4, 10, and 12 is, at least in part, due to perturbation of membrane stability.

The phenotypic switching of *C. albicans* between yeast and hyphal forms has been considered as one of the most significant virulent factors in *C. albicans*. Although some studies were conducted to evaluate the anti-candidal potential of benzothiazole compounds, very scarce publications addressed the anti-dimorphic potential of these compounds. The development of hyphae is an interesting property of *C. albicans* that plays a vital role in adherence and biofilm formation, which is certainly crucial for colonization and pathogenesis [35,36]. Thus, we hypothesized that the compounds that showed antifungal activity in our results would be able to inhibit hyphal transition in *C. albicans.* Our results showed that some benzothiazole compounds could inhibit the yeast-mycelial conversion at concentrations lower than their respective MIC values. Our findings are compatible with those of others [37] who reported similar ability of some benzothiazoles to selectively inhibit the mycelial form of five *C. albicans* strains at 100 µM. Similarly, the results obtained by Fabry et al. [38] showed that the toxic effect of 6-amino-2-*n*-pentylthiobenzothiazole on *C. albicans* was due to its ability to reduce hyphae formation in a concentration-dependent way.

Dihydroorotase is a zinc metalloenzyme that functions in the pathway for the biosynthesis of pyrimidine nucleotides by catalyzing the reversible interconversion of carbamoyl aspartate and dihydroorotate. The crystal structure of the dihydroorotase from *E. coli* was determined and it was found that the active site contains two zinc ions, which are bridged by a hydroxide group or a water molecule [39]. Structural studies confirmed the prediction that dihydroorotase is a member of the amidohydrolase superfamily with a (βα)8-barrel protein fold [39,40]. Several publications have investigated the role of inhibition of dihydroorotase in both Gram-positive and Gram-negative bacteria, which indicated its ubiquitous distribution and applicability as a suitable target for antimicrobial drug discovery. For instance, dihydroorotase was previously reported as an important drug target in the Gram-positive *B. anthracis* [41]. In addition, dihydroorotase was found to be essential for the survival of *S. aureus* [42]. Similar findings were also introduced in Gram-negative bacteria by others [40]. Moreover, this enzyme is equally essential in fungal cell metabolism [43]. However, the inhibitory effect of benzothiazole compounds on the activity of dihydroorotase is not well-understood. Our data showed that some of the studied benzothiazole compounds have potent inhibitory activity against *E.coli* dihydroorotase.

In further criticizing the obtained docking results in relation to the measured antimicrobial activity, the mode of binding of compounds with *E. coli* dihydroorotase was examined (Figure 4 and Figure 5). The standard ligand HDDP [39] formed an extensive hydrogen-bonding network with LEU222, ALA266, HIS254, ARG20, ASN44, and HIS139. Due to the larger size of benzothiazoles as compared with HDDP, the phenol group of compound 3 is lodged into the cavity hosting HDDP forming a single hydrogen bond with the side chain of LEU222. A similar finding was observed with compound 10, where the oxygen of the methoxy group formed a hydrogen bond with the side chain of ALA266. At the entrance of the catalytic cavity of *E. coli* dihydroorotase, the methylbenzothiazole of compound 3 forms extensive hydrophobic interactions with the side chains of ARG258, CYS256, and CYS221 and the naphthalene ring is also forming hydrophobic contacts with THR143, GLU141, and PRO105. The loss of hydrogen bonds due to the low number of potential hydrogen bonding donors or receptor groups accessing the receptor led to a lower estimated docking score. However, the presence of hydrophobic interactions and the bulky rings of thiazole and naphthalene at the entrance to the catalytic site might interfere with the access of substrates to the active site of dihydroorotase and contribute to the observed in vitro inhibition of the dihydroorotase enzyme, and, consequently, the estimated antimicrobial potency in the current study. Compound 3 showed a stacking interaction with HIS254. This residue was reported to be a vital element in the movement of the surface loop (residues 105–115), which stabilizes the transition state [39]. Furthermore, stacking of compound 3 with HIS254 may affect the transition state of the enzyme, which resulted in loss of activity.

These results provide evidence that the active benzothiazoles in the current study might be acting through inhibition of the dihydroorotase enzyme. However, one important limitation of our results is the lack of information on the possible contribution of other microbial targets, e.g., DNA gyrase, dihydropteroate synthase, and dihydrofolate reductase, which were previously identified as benzothiazole antimicrobial targets [18,19,20,21,22]. This can be a focus of future work.

In conclusion, some of the tested benzothiazoles in the current study showed promising antibacterial as well as moderate antifungal activities. Compounds 3, 4, 10, and 12 represent potential candidates as antimicrobial agents and add further evidence to the importance of the benzothiazole nucleus as a biologically active moiety. The mechanisms involved in the antimicrobial action of these compounds include, but are not limited to, disruption of membrane integrity and inhibition of dihydroorotase.

## 4. Materials and Methods

### 4.1. Biological Activity

All compounds were evaluated for in vitro antibacterial activities against *S. aureus*, *B. subtilis*, and *E. coli* strains and in vitro antifungal activity against *C. albicans* and *A. niger* strains by using the disc diffusion method. To study the probable mechanism, the effect of benzothiazole derivatives on the activity of *E. coli* dihydroorotase, morphogenesis of *C. albicans*, and leakage of DNA as well as proteins from *A. niger* was also evaluated.

#### 4.1.1. Antibacterial Activity of Benzothiazole Compounds

The antibacterial potential of benzothiazole derivatives was determined against three different bacterial strains previously used in our work [44]. The three strains include *S. aureus* (ATCC6538), *B. subtilis* (ATCC6438), and *E. coli* (ATCC8739) by the standard disc diffusion method as previously described [45,46]. The bacterial pathogens were maintained on nutrient agar media (Difco, Becton, Dickinson and Company, Sparks Glencoe, MD, USA). Prior to their use, the benzothiazole derivatives were prepared by dissolving the compounds in 5% DMSO and sonicating the samples at 30 °C for 15 min. Filter paper discs containing 50 µg/disc of each compound were used for the assay. Kanamycin at 5 µg/disc was used as a positive control, while discs impregnated in 5% DMSO only were used as the negative control. The overnight grown bacterial cultures (10^7^ colony-forming units/mL) were used. The antibacterial activity of the benzothiazole derivatives was determined by measuring the IZD after 24 h of incubation at 37 °C.

The MIC value for each of the benzothiazole derivatives was determined (final concentration range: 400–3.13 µg/mL prepared using a two-fold serial dilution) in test tubes containing 10 mL nutrient broth inoculated with the respective bacterial strain. Then, all the tubes were mixed properly and incubated at 37 °C overnight in a shaker incubator. Negative controls contained nutrient broth alone. The lowest concentration of each compound that did not show any visible growth of the respective test organism was determined as the MIC. To each test tube, 10 mL of the tested pathogen was added. This procedure was repeated three times for each compound for all the tested pathogens. For determination of the MBC, samples from test tubes containing the MIC concentration along with the next higher concentration were drug-free cultivated on nutrient agar plates for another 24 h at 37 °C. The concentration that did not show any growth of a single bacterial colony on the drug-free nutrient agar plates was defined as the MBC value [45,46]. Both MIC and MBC values were expressed in µg/mL.

#### 4.1.2. Antifungal Activity of Benzothiazole Compounds

The antifungal potential of the benzothiazole compounds in this study was compared to fluconazole, which is a standard antifungal agent, using the disc diffusion method, as previously described [47]. *C. albicans* and *A. niger* strains previously identified in our work [48,49] were used as model organisms in this experiment. Paper discs were prepared by adding 50 µg of each compound to a 6-mm filter paper disc. The fungi in liquid media (10^6^ colony-forming units/mL) were spread uniformly on potato-dextrose agar media (Difco, Becton, Dickinson and Company). The benzothiazole-containing or fluconazole-containing discs were placed on the plates, which were incubated at 28 °C for two days for *C. albicans* or at 25 °C for seven days for *A. niger*, respectively, before observation for fungal growth and formation of inhibition zones around the discs. The antifungal activity of each compound was determined by measuring the IZD around the respective paper disc. The MIC and minimum fungicidal concentration were determined as mentioned previously [47] and expressed in µg/mL.

#### 4.1.3. Effect of Benzothiazole Compounds on Dihydroorotase Activity

The dihydroorotase used in these assays was obtained from *E. coli* and purified as previously described [50]. The dihydroorotase ability to hydrolyze dihydroorotate and thio-dihydroorotate was monitored spectrophotometrically at 230 and 280 nm, respectively, as established previously [51] using SpectraMax (Molecular Devices, San Jose, CA, USA) plate reader. The assays were conducted in a 96-well quartz plate in a final volume of 250 µL potassium phosphate buffer (100 mM).

#### 4.1.4. Effect of Benzothiazole Compounds on *C. albicans* Hyphal Development in Liquid Media

Cultures of *C. albicans* grown overnight were incubated at 37 °C for 24 h with shaking in RPMI-1640 medium to induce hyphal development. The medium was supplemented with the benzothiazole compounds. RPMI-1640 medium without any antifungal agent was used as a negative control. Aliquots of fungal cells were harvested after 24 h and examined using bright-field microscopy with the help of a Digital Cell Imaging System (Logos Biosystems, Gyeonggi-do, South Korea) [52].

#### 4.1.5. Effect of Benzothiazole Compounds on the Leakage of DNA and Proteins

The effect of benzothiazole derivatives on the leakage of DNA and proteins from *A. niger* spores was determined by measuring the OD of the spore supernatant at 260 and 280 nm, respectively. This process was performed according to a method described by Khalil et al. [53] with slight modifications. In brief, the spore suspension (1 × 10^6^ spores/mL) was prepared by incubating *A. niger* at 28 °C for 24 h in potato-dextrose broth medium supplemented with four different concentrations of the most effective benzothiazole compounds under study. The potato-dextrose broth medium without any compound was used as a negative control.

### 4.2. Computational Studies

*E. coli* dihydroorotase has been described as a molecular target for benzothiazoles [23]. Computational and molecular modeling studies were carried out to explain the molecular basis underlying the interaction of benzothiazole derivatives with *E. coli* dihydroorotase. The crystal structure of *E. coli* dihydroorotase was retrieved from the protein data bank (PDB ID 2eg7). The standard *E. coli* dihydroorotase ligand HDDP was used for comparison. The protein structure was prepared and optimized by the protein preparation module in the Maestro software package, version 12 (Schrödinger, New York, NY, USA). The water molecules and other crystal-bound molecules were removed. The protein was protonated and optimized at physiological pH conditions. Energy minimization of the protein was carried out by the OPLS2005 force field. The docking grid was centered around the co-crystalized ligand in a box size of 15 Å. The structures of the compounds were 3D-optimized using OPLS2005 force field by the Ligprep module in the Schrödinger suite. The Schrödinger glide docking module was used for docking the compounds into the *E. coli* dihydroorotase. The docking scores were implemented by using the standard precision (SP) docking mode.

### 4.3. Statistical Analysis

The results of all the experiments were expressed as the mean value of three independent replicates ± standard deviation. Statistical analysis of the significant differences between the mean values of the results was identified by one-way analysis of variance (ANOVA), which was followed by Duncan’s test at the 5% level of significance (*p* < 0.05) using the Statistical Analysis Software (SAS) package (Version: SAS9.4, SAS Institute Inc., Cary, NC, USA).

## Figures and Tables

**Table 1:** Antimicrobial activity of tested compounds against some pathogenic strains.

Compound	Fungi						Gram-Positive Bacteria						Gram-Negative Bacteria		
	*Candida albicans*			*Aspergillus niger*			*Staphylococcus aureus*			*Bacillus subtilis*			*Escherichia coli*		
	IZD (mm)	MIC (µg/mL)	MFC (µg/mL)	IZD (mm)	MIC (µg/mL)	MFC (µg/mL)	IZD (mm)	MIC (µg/mL)	MBC (µg/mL)	IZD (mm)	MIC (µg/mL)	MBC (µg/mL)	IZD (mm)	MIC (µg/mL)	MBC (µg/mL)
**1**	0 ^f^ ± 0.0	-	-	9 ^e^ ± 0.12	200 ^a^	400 ^a^	13 ^e^ ± 0.53	100 ^b^	200 ^b^	15 ^d^ ± 0.32	200 ^a^	400 ^a^	0 ^h^ ± 0.0	-	-
**2**	9 ^e^ ± 0.27	100 ^a^	200 ^a^	7 ^f^ ± 0.15	200 ^a^	400 ^a^	15 ^d^ ± 0.31	50 ^c^	100 ^c^	14 ^e^ ± 0.12	100 ^b^	200 ^b^	6 ^f^ ± 0.11	100 ^a^	200 ^a^
**3**	15 ^c^ ± 0.38	25 ^c^	50 ^c^	18 ^b^ ± 0.91	25 ^d^	50 ^d^	25 ^b^ ± 0.24	50 ^c^	100 ^c^	21 ^c^ ± 0.31	25 ^d^	50 ^d^	27 ^b^ ± 0.25	25 ^c^	50 ^c^
**4**	18 ^b^ ± 0.54	50 ^b^	100 ^b^	12 ^d^ ± 0.23	50 ^c^	100 ^c^	19 ^c^ ± 0.61	50 ^c^	100 ^c^	22 ^b^ ± 0.26	50 ^c^	100 ^c^	25^c^ ± 0.31	50 ^b^	100 ^b^
**5**	0 ^f^ ± 0.0	-	-	0 ^g^ ± 0.0	-	-	10 ^a^ ± 0.52	200 ^a^	400 ^a^	14 ^e^ ± 0.57	200 ^a^	400 ^a^	18 ^d^ ± 0.51	100 ^a^	200 ^a^
**6**	0 ^f^ ± 0.0	-	-	0 ^g^ ± 0.0	-	-	0 ^f^ ± 0.0	-	-	0 ^f^ ± 0.0	-	-	0 ^g^ ± 0.0	-	-
**7**	0 ^f^ ± 0.0	-	-	0 ^g^ ± 0.0	-	-	0 ^f^ ± 0.0	-	-	0 ^f^ ± 0.0	-	-	0 ^g^ ± 0.0	-	-
**8**	0 ^f^ ± 0.0	-	-	0 ^g^ ± 0.0	-	-	0 ^f^ ± 0.0	-	-	0 ^f^ ± 0.0	-	-	0 ^g^ ± 0.0	-	-
**9**	0 ^f^ ± 0.0	-	-	0 ^g^ ± 0.0	-	-	0 ^f^ ± 0.0	-	-	0 ^f^ ± 0.0	-	-	0 ^g^ ± 0.0	-	-
**10**	13 ^d^ ± 0.11	100 ^a^	200 ^a^	11 ^b^ ± 0.02	100 ^b^	200 ^b^	15 ^d^ ± 0.21	50 ^c^	100 ^c^	15 ^d^ ± 0.23	50 ^c^	100 ^c^	12 ^e^ ± 0.61	25 ^c^	50 ^c^
**11**	0 ^f^ ± 0.0	-	-	0 ^g^ ± 0.0	-	-	0 ^f^ ± 0.0	-	-	0 ^f^ ± 0.0	-	-	0 ^g^ ± 0.0	-	-
**12**	16 ^c^ ± 0.21	50 ^b^	100 ^b^	14 ^c^ ± 0.36	100 ^b^	200 ^b^	20 ^c^ ± 0.06	100 ^b^	200 ^b^	14 ^e^ ± 0.15	100 ^b^	200 ^b^	19 ^d^ ± 0.19	50 ^b^	100 ^b^
**13**	0 ^f^ ± 0.0	-	-	0 ^g^ ± 0.0	-	-	0 ^f^ ± 0.0	-	-	0 ^a^ ± 0.0	-	-	0 ^g^ ± 0.0	-	-
**DMSO**	0 ^f^ ± 0.0	-	-	0 ^g^ ± 0.0	-	-	0 ^f^ ± 0.0	-	-	0 ^f^ ± 0.0	-	-	0 ^g^ ± 0.0	-	-
**Fluconazole**	28 ^a^ ± 0.62	6.25 ^d^	12.5 ^d^	26 ^a^ ± 0.82	3.13 ^e^	6.25 ^e^	0 ^f^ ± 0.0	-	-	0 ^f^ ± 0.0	-	-	0 ^g^ ± 0.0	-	-
**Kanamycin**	0 ^f^ ± 0.0	-	-	0 ^g^ ± 0.0	-	-	29 ^a^ ± 0.84	6.25 ^d^	12.5 ^d^	28 ^a^ ± 0.62	3.13 ^d^	6.25 ^e^	31 ^a^ ± 0.81	6.25 ^d^	12.5 ^d^

The means ± standard deviation followed by the same superscript letter in the same column are not significantly different according to ANOVA and Duncan’s multiple range tests at *p* < 0.05. IZD: inhibition zone diameter, MIC: minimum inhibitory concentration, MFC: minimum fungicidal concentration, MBC: minimum bactericidal concentration, DMSO: Dimethyl sulfoxide, (-): no activity.

**Table 2:** Effect of tested compounds on specific activities of dihydroorotase enzyme of *Escherichia coli*.

Compound	Specific Activity of *Escherichia coli* Dihydroorotase (nmol/min/mg protein)
**DMSO**	119 ^a^ ± 1.7
**Kanamycin**	26 ^l^ ± 0.38
**1**	85 ^f^ ± 0.91
**2**	100 ^c^ ± 1.1
**3**	45 ^k^ ± 0.62
**4**	60 ^j^ ± 0.015
**5**	82 ^g^ ± 1.15
**6**	92 ^e^ ± 1.51
**7**	111 ^b^ ± 0.98
**8**	98 ^d^ ± 0.82
**9**	102 ^c^ ± 0.91
**10**	65 ^i^ ± 0.43
**11**	73 ^h^ ± 0.24
**12**	75 ^h^ ± 0.31
**13**	104 ^c^ ± 0.73

The means ± standard deviation followed by the same superscript letter in the same column are not significantly different, according to ANOVA and Duncan’s multiple range tests at *p* < 0.05.

**Table 3:** Effect of different benzothiazole compounds on the dimorphic transition of *Candida albicans*.

Compound	Yeast Form Count (cell/mL)	Filamentous Form Count (cell/mL)	% of Dimorphism
**Control (DMSO)**	50 ^k^ ± 4.0	1730 ^e^ ± 4.0	97.1
**Fluconazole**	350	400 ^k^ ± 0.5	12.5
**1**	410 ^d^ ± 5.0	1810 ^d^ ± 1.0	77.3
**2**	450 ^b^ ± 2.5	1178 ^i^ ± 1.5	61.7
**3**	306 ^e^ ± 1.0	393 ^l^ ± 1.5	22
**4**	10 ^l^ ± 2.5	12 ^o^ ± 0.76	16.66
**5**	448 ^c^ ± 5.0	1320 ^h^ ± 5.0	66
**6**	162 ^i^ ± 2.5	2033 ^a^ ± 1.5	92
**7**	186 ^g^ ± 4.5	1691 ^f^ ± 0.5	89
**8**	162 ^i^ ± 5.0	1620 ^g^ ± 2.5	90
**9**	92 ^j^ ± 7.5	1840 ^c^ ± 2.0	95
**10**	243 ^f^ ± 5.0	363 ^m^ ± 1.5	33
**11**	10 ^l^ ± 2.5	144 ^n^ ± 2.0	93
**12**	497 ^a^ ± 6.0	904 ^j^ ± 2.0	45
**13**	164 ^h^ ± 7.5	1844 ^b^ ± 2.0	92.10

% of dimorphism = (Filamentous form count - Yeast form count)/Filamentous form count × 100. The means ± standard deviation followed by the same superscript letter in the same column are not significantly different, according to ANOVA and Duncan’s multiple range tests at *p* < 0.05. DMSO: Dimethyl sulfoxide.

**Table 4:** The docking scores, ligand efficiency, and interaction parameters of benzothiazole compounds with *Escherichia coli* dihydroorotase.

Compound	Docking Score	Glide Ligand Efficiency	Glide Lipo	Glide H-Bond
**HDDP**	−7.37	−0.57	−0.15	−0.80
**3**	−5.02	−0.17	−1.70	−0.47
**4**	−4.57	−0.16	−1.84	−0.32
**10**	−4.87	−0.16	−1.61	−0.52
**12**	−2.54	−0.08	−0.97	−0.16

HDDP: 2-oxo-1,2,3,6-tetrahydropyrimidine-4,6-dicarboxylic acid.
